# Neoadjuvant chemotherapy weakens the prognostic value of the pathological tumor burden score for colorectal cancer liver metastases

**DOI:** 10.1186/s12893-023-02145-w

**Published:** 2023-09-09

**Authors:** Leen Liao, Hui Sun, Jiahua He, Yujun Liu, Zhizhong Pan, Xiaojun Wu, Wenhua Fan, Jianhong Peng, Cong Li

**Affiliations:** 1 Department of Colorectal Surgery, State Key Laboratory of Oncology in South China, Collaborative Innovation Center for Cancer Medicine, Sun Yat-sen University Cancer Center, Guangzhou, Guangdong 510060 P. R. China; 2 Zhongshan School of Medicine, Sun Yat-sen University, Guangzhou, Guangdong 510060 P. R. China

**Keywords:** Colorectal Cancer Liver Metastases, Pathological tumor burden score, Neoadjuvant Chemotherapy

## Abstract

**Background:**

The pathological tumor burden score (TBS) has been proven to be a better risk stratification tool for liver metastasis of colorectal cancer than the traditional clinical risk score (CRS). The aim of this study was to evaluate the prognostic value of the pathological tumor burden score in patients with or without neoadjuvant chemotherapy (NAC).

**Methods:**

A total of 348 patients with colorectal liver metastases (CRLM) who underwent curative hepatic resection were retrospectively enrolled from September 1999 to December 2016. Univariable and multivariable Cox regression analyses were conducted to identify the independent predictors of prognosis. Kaplan–Meier curves and log-rank tests were used to determine whether TBS has enough discriminatory ability under certain grouping.

**Results:**

Patients who received NAC had a higher median TBS than patients who did not receive NAC (4.07 vs. 2.69, *P* < 0.001). Among patients who did not receive NAC, those with TBS > 3 showed a significantly worse 3-year RFS (41.1% vs. 63.6%, *P* < 0.001) and 3-year OS rate (73.3% vs. 84.1%, *P* = 0.003) than those with TBS ≤ 3. Among the patients who received NAC, those with TBS ≤ 3 or TBS > 3 showed comparable 3-year RFS (33.3% vs. 26.4%, *P* = 0.400) and 3-year OS rates (76.5% vs. 58.2%, *P* = 0.064) to those who did not. Regardless of the regimen and response to NAC, there was no significant difference about 3-year RFS and 3-year OS rates between the TBS ≤ 3 and TBS > 3 groups.

**Conclusion:**

Pathological TBS can be applied to predict the RFS and OS of patients suffering from CRLM who did not receive NAC. However, pathological TBS might not be regard as prognosis in patients who did receive NAC.

## Introduction

Estimated 1.8 million people worldwide had colorectal cancer (CRC) in 2018 [1]. Metastatic sites included lung, liver, bone, lymph node, and peritoneum, with liver being the most common metastatic site [2]. For patients with colorectal liver metastases (CRLM), different strategies should be applied according to the severity of the disease, physical conditions, etc. Although hepatic resection remains the most effective curative-intent treatment for patients with CRLM, over half of patients will develop recurrent disease within two years after liver resection [3]. Numerous traditional scoring criteria helps us on stratifying the patient and accurately evaluate their prognosis [4–8]. Among these scoring systems, only the Memorial Sloan-Kettering Cancer Center Clinical Risk Score (MSKCC-CRS) and the Iwatsuki score showed statistically significant stratification capacity on survival [9]. However, with the introduction of neoadjuvant chemotherapy (NAC), the level of the clinicopathological index of patients who received NAC may change significantly, including the reduced size and number of pathological CRLM, which are parts of several traditional scoring criteria. Studies have shown that many traditional scoring criteria, including MSKCC-CRS, did not predict clinical prognosis of CRLM patients who received NAC [10].

Kazunari Sasaki et al. first established the tumor burden score (TBS) by combining the size and number of liver metastases obtained from pathological specimens, and it was proven to have higher values to predict the overall survival (OS) of patients with CRLM than the clinical risk score (CRS) system [11]. Recently, Chen et al. [12] improved the TBS system for Chinese patients and proposed the Comprehensive Evaluation of Relapse Risk (CERR) to help determine optimal clinical management strategies. However, for patients receiving NAC, the volume and number of tumors may significantly change by comparison of tumors at baseline. Therefore, TBS might not actually reflect tumor burden for the patients. Up to date, little is known in the clinical significance of pathological TBS in cases who receive NAC. Whether pathological TBS could effectively predict the long-term survival of CRLM patients remains unclear.

Therefore, this study intends to evaluate the prognostic value of pathological TBS in patients with or who did not receive NAC to identify the specific conditions under which TBS can be used.

## Material and method

### Patients and data collection

We reviewed clinical data from 348 consecutive CRLM patients who received resection of the primary tumor and liver metastases from September 1999 to December 2016 at Sun Yat-sen University Cancer Center. All patients meeting the following criteria were eligible for inclusion: (1) histologically diagnosed with colorectal adenocarcinoma, (2) metastases limited to the liver, (3) radical resection for both the primary tumor and liver metastases, and (4) follow-up period after liver resection of at least 3 months. Clinical information and follow-up results of the patients was obtained by using an electronic medical record system at Sun Yat-sen University Cancer Center. Informed consent was obtained from all patients whose clinical data were used. The study was approved by the Institutional Research Ethics Committee of Sun Yat-sen University Cancer Center (approval number: B2020-301-01).

### Parameter measurements and cut off value

Patients were histologically evaluated according to the eighth edition of American Joint Committee on Cancer (AJCC) staging system. Enhanced abdominal magnetic resonance imaging (MRI) or computed tomography (CT) was used to evaluate the characteristics of liver metastases, including number, diameter, and distribution before liver resection. Pathological TBS was calculated by using the following mathematical equation: (TBS)^2^= (pathological maximum liver tumor diameter in cm)^2^ + (number of tumors)^2^. We defined the cut-off value of TBS as 3 according to a previous study^11^. Synchronous metastasis was defined as liver metastasis diagnosed before colorectal resection or at the time of surgery [13].

Tumor biomarkers such as carcinoembryonic antigen (CEA) and cancer antigen 19 − 9 (CA19-9) have been detected before hepatectomy. The cut-off values of CEA and CA19-9 levels were 5 ng/ml and 35 U/ml, respectively, according to our previous study [14, 15]. The therapeutic strategy applied to patients with synchronous liver metastases were determined based on consensus by multidisciplinary team (MDT), which is recommended by Nordlinger’s study [16]. Recurrence risk in patients was evaluated by the MSKCC-CRS [7]. Patients with an MSKCC-CRS of 3–5 were classified into the high-risk subgroup and recommended for NAC .

### Follow up

After hepatectomy, patients were followed up every 3 months for the first 2 years and then semiannually until 5 years. During each clinical review, blood levels of CEA and CA19-9 were measured, and CT imaging of the chest, abdomen and pelvis was performed at 3, 6, 12, 18 and 24 months, then annually thereafter. Liver MRI was used to identify suspicious lesions shown on CT or cases with negative CT results and elevated levels of CEA or CA19-9. The diagnosis of recurrence was typically made based on an integrated assessment of all available information, including imaging results, clinical symptoms, and pathological findings if available.Overall survival (OS) was calculated from hepatectomy to death of any cause or the last follow-up, while recurrence-free survival (RFS) was defined as the interval between hepatectomy and recurrence, death or last follow-up. The last follow-up visit took place in February 2020.

### Statistical analysis

Statistical analyses were performed using IBM SPSS Statistics 24 software (IBM, NY, USA), GraphPad Prism version 6.01 (GraphPad Software, Inc., USA) and R software packages.

Values are presented as the median (range) and percentage. DFS and OS was compared using the Kaplan-Meier method with log-rank test. Parameters showing statistical significance for OS and RFS in univariate Cox models were further assessed using multivariate Cox models. Hazard ratios (HRs) and 95% CIs were subsequently calculated. All statistical tests used in this study were two-sided, and a *P* value < 0.05 was considered statistically significant.

## Results

### Patient characteristics and TBS distribution

**Fig. 1:**
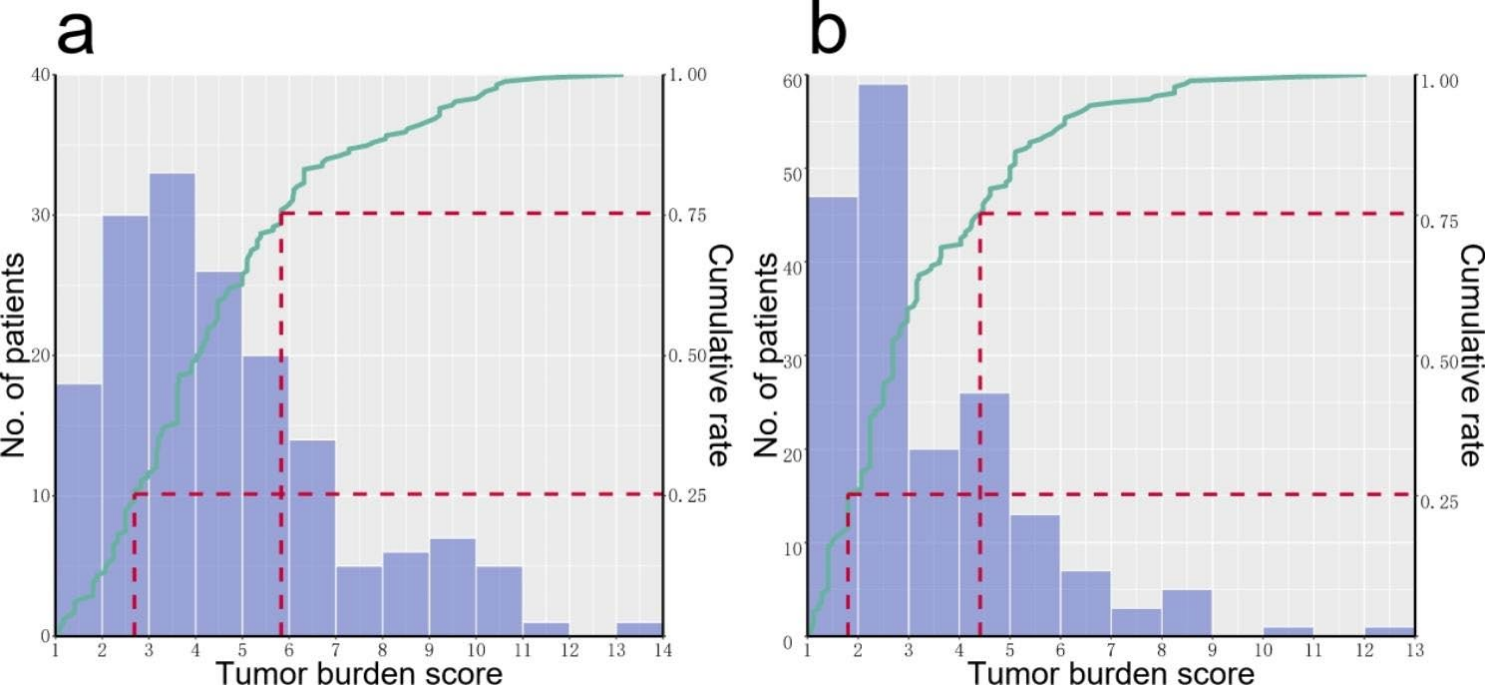
The distribution of the tumor burden score (TBS) in patients with and without neoadjuvant chemotherapy. **a**, The distribution of TBS in patients with neoadjuvant chemotherapy. **b**, The distribution of TBS in patients without neoadjuvant chemotherapy

Patient characteristics are summarized in Table 1. Of the 348 patients, 231 (66.4%) were male, and 117 (33.6%) were female, with a median age of 57.48 [interquartile range (IQR) 49.42–64.46]. Overall, 166 (47.7%) patients received NAC, among whom 110 (66.3%) received oxaliplatin-based chemotherapy, 45 (27.1%) received irinotecan-based chemotherapy, and 11 (6.6%) received other chemotherapy regimens. In addition, 245 (70.4%) patients received postoperative chemotherapy. The median TBS was 3.2 (IQR 2.24–5.10), and TBS = 3 was regarded as the dividing point. Thus, 154 (44.3%) patients were classified into the low-TBS (TBS ≤ 3) group, and 194 (55.7%) were classified into the high-TBS (TBS > 3) group. For the patients who received NAC, the median TBS was 4.07 (IQR 2.69–5.83), the maximum TBS was 13.15, and the minimum TBS was 1.08 (Fig. 1a). For the patients who did not receive NAC, the median TBS was 2.69 (IQR 1.8-4.425), the maximum TBS was 12.04, and the minimum TBS was 1.04 (Fig. 1b). Obviously, patients who received NAC showed a right-shift distribution of TBS compared with patients who did not receive NAC. Independent sample Mann-Whitney U test showed that the average rank of patients who did not undergo NAC was 146.21, and that of the patients who received NAC was 205.52, with a P value less than 0.001, which means that the total TBS score in patients who received NAC was higher than that in patients who did not receive NAC.

**Table 1:** Clinicopathologic characteristics of 348 consecutive CRLM patients who underwent primary tumor

Characteristics	No. of patients (%)
Patient characteristics	
Median age (years)	57(49–64)
Gender	
Male Female	231(66.4) 117(33.6)
Primary tumor location	
Colon Rectum	220(63.2) 128(36.8)
T stage	
T1-3 T4	209(60.1) 139(39.9)
Nodal metastases	
Positive Negative	216(62.1) 132(37.9)
Primary tumor differentiation	
Well to moderate Poor	264(75.9) 84(24.1)
Timing of metastasis	
Synchronous Metachronous	239(68.7) 109(31.3)
RECIST response^a^	
SD or PD PR	61(37.7) 101(62.3)
Preoperative CEA (ng/mL)^b^	
≤ 5 > 5	131(39.8) 198(60.2)
Preoperative CA19-9 (U/ml)^c^	
≤ 35 > 35	221(69.1) 99(30.9)
Number of liver metastases, median (IQR)	2(1–3)
Size of largest liver metastasis(cm), median(IQR)	2.5(1.5–3.8)
TBS, median(IQR)	3.2(2.2–5.1)
Tumor distribution	
Unilobar	245
Bilobar	103
KRAS mutation status^d^	
Wild-type Mutation-type	55 34
Postoperative chemotherapy	
Yes No	245 103

CRLMs, colorectal liver metastases; TNM stage, tumor-node-metastasis classification; IQR, interquartile range; CEA, carcinoembryonic antigen; CA19-9, carbohydrate antigen 19 − 9; TBS, tumor burden score; RECIST, Response Evaluation Criteria in Solid Tumor; SD, stable disease; PD, progressive disease; PR, partial response;

^a^ Data were available for 162 patients

^b^ Data were available for 329 patients

^c^ Data were available for 320 patients

^d^ Data were available for 89 patients

### Relationship between receiving neoadjuvant therapy and patient characteristics

As shown in Table 2, compared with patients who did not receive NAC, a larger proportion of patients who received NAC underwent postoperative chemotherapy (75.9% vs. 65.4%, *P* = 0.032), were of a younger age (69.3% vs. 53.8%, *P* = 0.003), and had T4 stage disease (45.8% vs. 34.6%, *P* = 0.034), synchronous CRLM tumor (78.3% vs. 59.9%, *P <* 0.001), more than one CRLM tumor (71.7% vs. 31.3%, *P* < 0.001), a higher TBS score (71.1% vs. 41.8%, *P* < 0.001), and bilobar disease (45.2% vs. 15.4%, *P* < 0.001).

**Table 2:** Clinicopathologic characteristics of all patients stratified by neoadjuvant chemotherapy

Characteristics	Neoadjuvant chemotherapy (n = 166)	Without neoadjuvant chemotherapy (n = 182)	*P* value
Age, years			0.003
≤ 60 > 60	115(69.3) 51(30.7)	98(53.8) 84(46.2)	
Gender			0.187
Male Female	116(69.9) 50(30.1)	115(63.2) 67(36.8)	
Primary tumor location			0.199
Colon Rectum	99(59.6) 67(40.4)	121(66.5) 61(33.5)	
T stage			0.034
T1- T3 T4	90(54.2) 76(45.8)	119(65.4) 63(34.6)	
Nodal metastases			0.120
Positive Negative	70(42.2) 96(57.8)	62(34.1) 120(65.9)	
Primary tumor differentiation			0.462
Well to moderate Poor	123(74.1) 43(25.9)	141(77.5) 41(22.5)	
Timing of metastasis			< 0.001
Synchronous Metachronous	130(78.3) 36(21.7)	109(59.9) 73(40.1)	
Preoperative CEA (ng/ml)^a^			0.251
≤ 5 > 5	68(43) 90(57)	63(43) 108(57)	
Preoperative CA19-9 (U/ml)^b^			0.145
≤ 35 > 35	111(73) 43(27)	110(65.5) 58(31.9)	
Number of CRLM			< 0.001
1 > 1	47(28.3) 119(71.7)	125(68.7) 57(31.3)	
Size of largest CRLM (cm)			0.063
≤ 3 > 3	104(62.7) 62(37.3)	131(72) 51(28)	
TBS			< 0.001
≤ 3 > 3	48(28.9) 118(71.1)	106(58.2) 76(41.8)	
Tumor distribution			< 0.001
Unilobar Bilobar	91(54.8) 75(45.2)	154(84.6) 28(15.4)	
Postoperative chemotherapy			0.032
Yes No	126(75.9) 40(24.1)	119(65.4) 63(34.6)	

CEA, carcinoembryonic antigen; CA19-9, carbohydrate antigen 19 − 9; CRLMs, colorectal liver metastases; TBS, tumor burden score

^a^ Data were available for 329 patients

^b^ Data were available for 322 patients

### Survival analysis

With a median follow-up time of 39 months (IQR 25–58 months), 119 (34.2%) patients were alive and disease-free, 60 (17.2%) patients were alive with tumor recurrence, and 169 (48.6%) patients experienced cancer-related mortality. The 3-year RFS rate and OS rate were 36.8% and 58.3%, respectively.

**Fig. 2:**
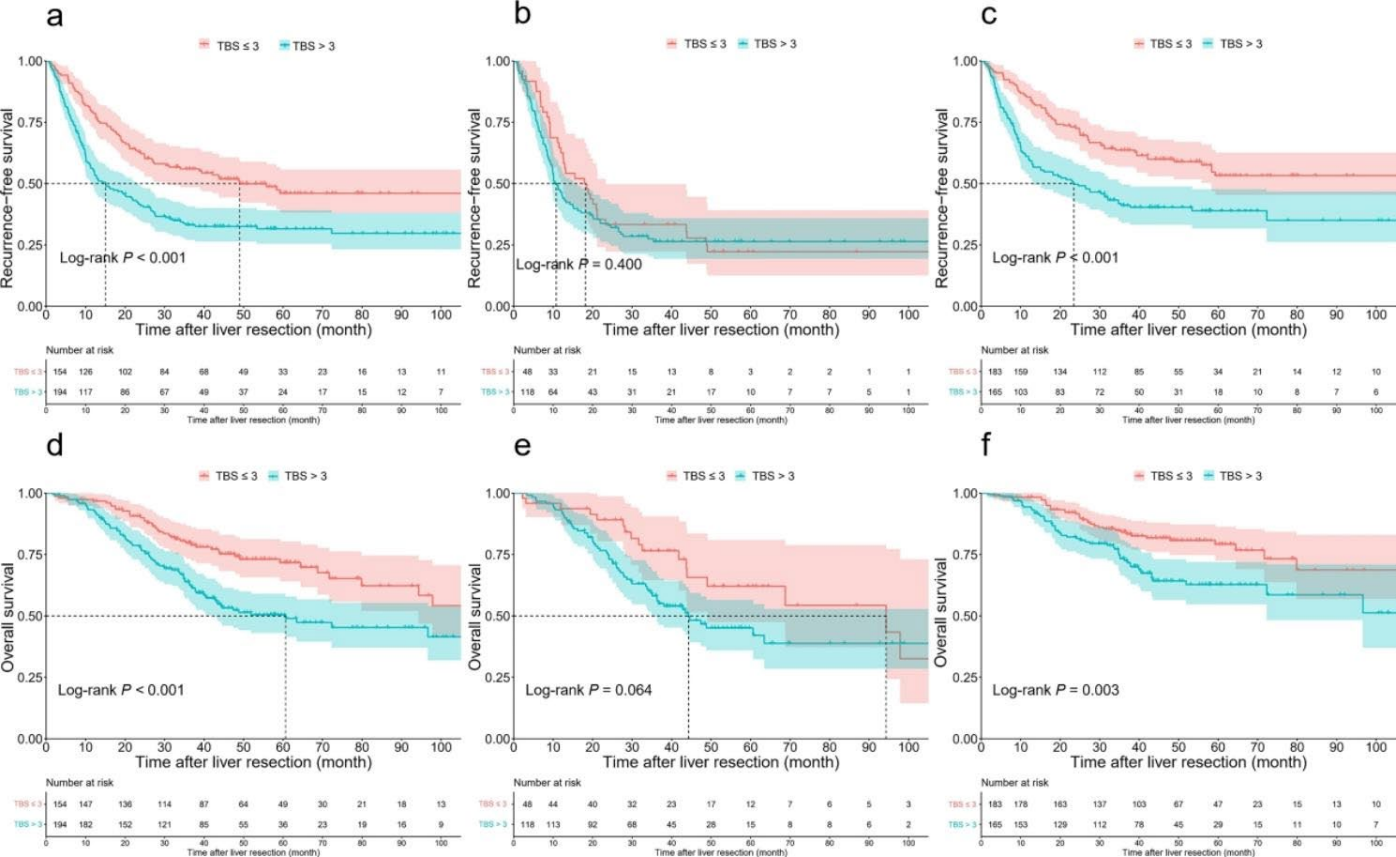
Comparison of recurrence-free survival (RFS) and overall survival (OS) after curative liver resection among all patients and patients with or without neoadjuvant chemotherapy (NAC) according to tumor burden score (TBS). **a**, Comparison of RFS in the low and high TBS groups among all patients. **b**, Comparison of RFS in the low and high TBS groups among patients who received NAC. **c**, Comparison of RFS in the low and high TBS groups among patients who did not receive NAC. **d**, Comparison of OS in the low and high TBS groups among all patients. **e**, Comparison of OS in the low and high TBS groups among patients who received NAC. **f**, Comparison of OS in the low and high TBS groups among patients who did not receive NAC.

**Fig. 3:**
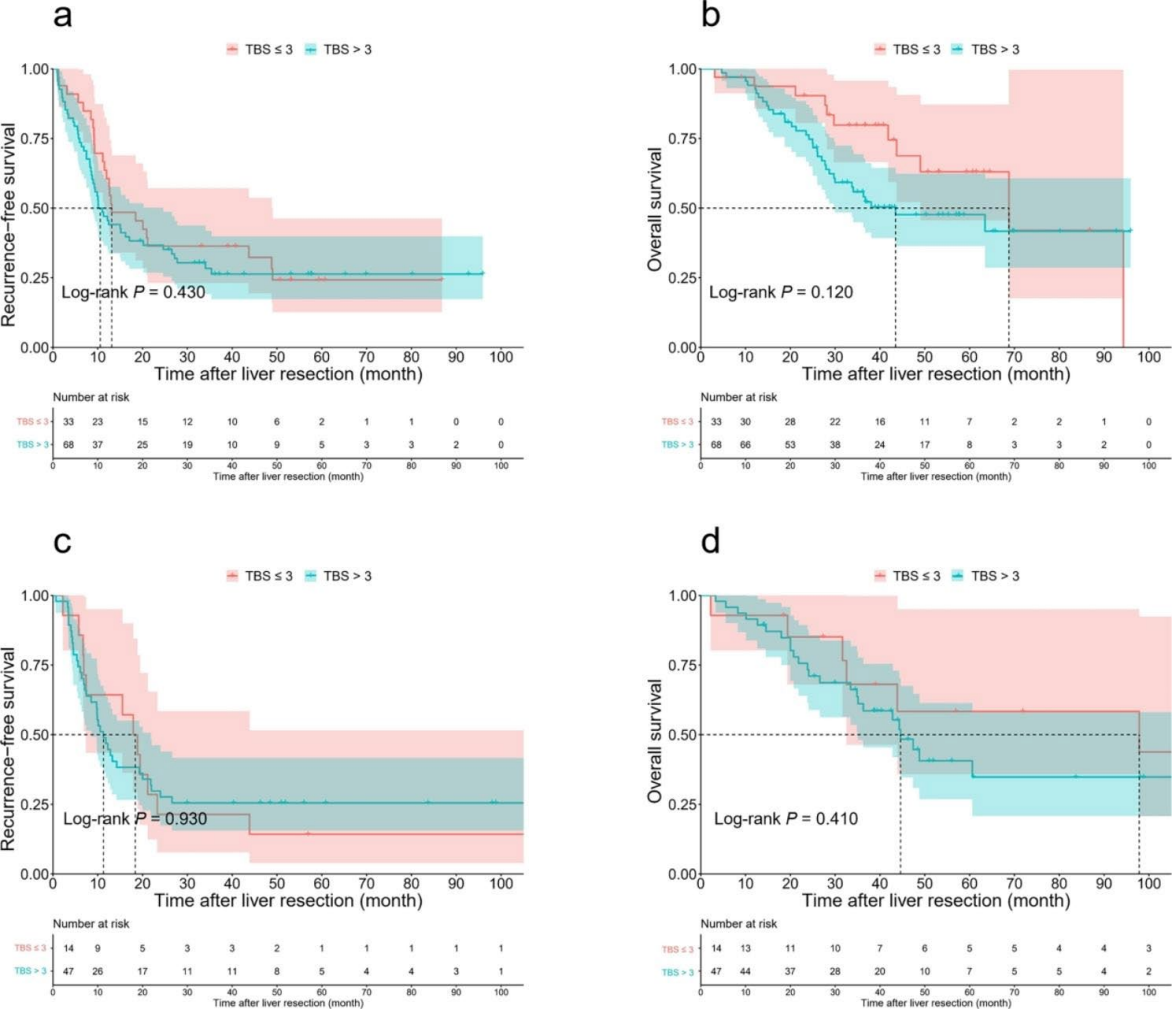
Comparison of recurrence-free survival (RFS) and overall survival (OS) after curative liver resection for patients who received neoadjuvant therapy stratified by response to NAC. **a**, Comparison of RFS in the low and high TBS groups among patients with partial response (PR). **b**, Comparison of OS in the low and high TBS groups among patients with PR. **c**, Comparison of RFS in the low and high TBS groups among patients with stable disease (SD) or progressive disease (PD). **d**, Comparison of OS in the low and high TBS groups among patients with SD or PD

Patients with TBS ≤ 3 showed a significantly higher 3-year RFS rate than those with TBS > 3 [56% (95% CI, 48.6–64.5%) vs. 33.2% (95% CI, 27.1–40.7%), *P* < 0.001] (Fig. 2a). Similarly, patients with TBS ≤ 3 showed a significantly higher 3-year OS rate than those with TBS > 3 [79.7% (95% CI, 73.4–86.6%) vs. 63.8% (95% CI, 57.2–71.3%), *P* < 0.001] (Fig. 2d). Among the patients who received NAC, patients with either TBS ≤ 3 or TBS > 3 presented with comparable 3-year RFS rates [33.3% (95% CI, 22.3–49.7%) vs. 26.4% (95% CI, 19.4–35.9%); *P* = 0.400, Fig. 2b] and 3-year OS rates [76.5% (95% CI, 64.7–90.5%) vs. 58.2% (95% CI, 49.7–68.1%); *P* = 0.064, Fig. 2e]. Among the patients who did not receive NAC, patients with TBS ≤ 3 had a significantly higher 3-year RFS rate than those with TBS > 3 [63.6% (95% CI, 56.9–71.1%) vs. 41.1% (95% CI, 34.1–49.6%), *P* < 0.001] (Fig. 2c). Similarly, patients with TBS ≤ 3 had a significantly higher 3-year OS rate than those with TBS > 3 [84.1% (95% CI, 78.7–89.8%) vs. 73.3% (95% CI, 66.5–80.8%), *P* = 0.003] (Fig. 2f).

**Fig. 4:**
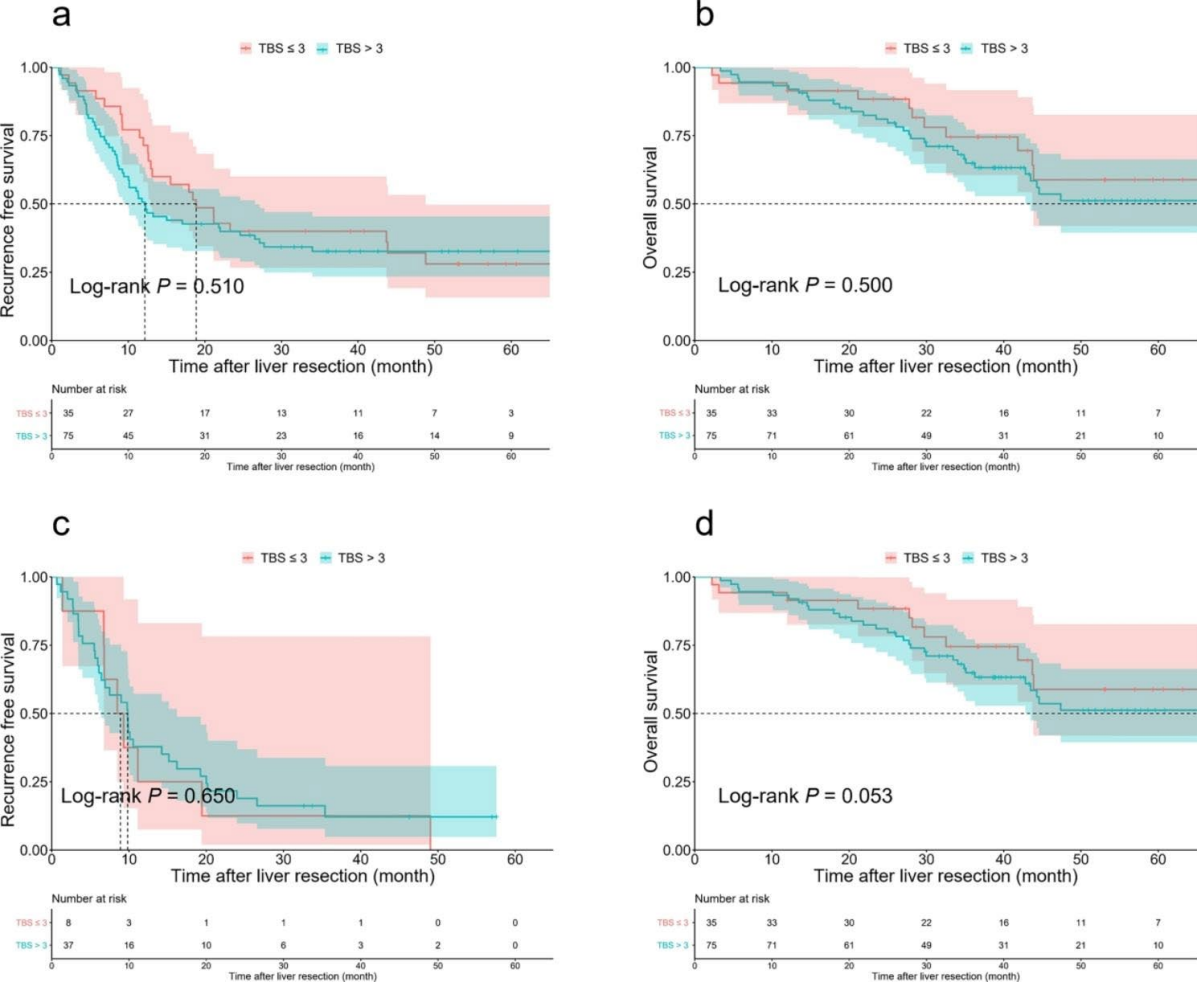
Comparison of recurrence-free survival (RFS) and overall survival (OS) after curative liver resection for patients who received neoadjuvant therapy stratified by NAC regimen. **a**, Comparison of RFS in the low and high TBS groups among patients with oxaliplatin-based NAC. **b**, Comparison of OS in the low and high TBS groups among patients with oxaliplatin-based NAC. **c**, Comparison of RFS in the low and high TBS groups among patients with irinotecan-based NAC. **d**, Comparison of OS in the low and high TBS groups among patients with irinotecan-based NAC.

Among the patients who received NAC, the 3-year RFS rates of patients with TBS ≤ 3 and TBS > 3 were comparable for both those who had PR (36.4% vs. 26.3%, *P* = 0.430, Fig. 3a) and those who had PD or SD (21.4% vs. 25.5%, *P* = 0.930, Fig. 3c). Similarly, the 3-year OS rates of patients with TBS ≤ 3 and TBS > 3 were also comparable for both those who had PR (79.8% vs. 55.8%, *P* = 0.120, Fig. 3b) and those who had PD or SD (68.1% vs. 61.1%, *P* = 0.410, Fig. 3d). For patients who received oxaliplatin-based chemotherapy, the 3-year RFS and OS rates of patients with TBS ≤ 3 and TBS > 3 were not significantly different (RFS: 40.0% vs. 32.6%, *P* = 0.510, Fig. 4a; OS: 74.5% vs. 64.9%, *P* = 0.500, Fig. 4b). For patients who received irinotecan-based chemotherapy, the 3-year RFS and OS rates of patients with TBS ≤ 3 and TB S > 3 were not significantly different (RFS: 12.5% vs. 12.2%, *P* = 0.650, Fig. 4c; OS: 85.7% vs. 45.3%, *P* = 0.053 Fig. 4d).

### Univariate and multivariate analyses of the prognostic factors

For patients who received NAC, the univariate and multivariate Cox analyses of RFS and OS are summarized in Table 3. Univariate analysis revealed that positive nodal metastases and postoperative adjuvant therapy were associated with unfavourable RFS. Multivariate analysis showed that positive nodal metastases (HR 1.899; 95% CI 1.298–2.748; *P* = 0.001) and postoperative adjuvant therapy (HR 1.578; 95% CI 1.057–2.356; *P* = 0.026) were also independent predictive factors for unfavourable RFS. The univariate analysis revealed that only bilobar disease (HR 1.652; 95% CI 1.048–2.603; *P* = 0.031) was associated with unfavourable OS.

**Table 3:** Univariate and multivariate analyses of prognostic factors for overall survival in patients with neoadjuvant chemotherapy

Characteristics	RFS				OS	
	Univariable		Multivariable		Univariable	
	HR(95% CI)	*P* value	HR(95% CI)	*P* value	HR(95% CI)	*P* value
Age(> 60 vs. ≤60 years)	1.184(0.813–1.724)	0.378			1.398(0.8869–2.251)	0.168
Sex (male vs. female)	1.293(0.863–1.936)	0.213			0.788(0.472–1.313)	0.360
Primary tumor location (rectum vs. colon)	1.037(0.725–1.484)	0.841			1.146(0.725–1.81)	0.560
Primary T stage (4 vs. 1–3)	0.999(0.700-1.427)	0.998			0.909(0.579–1.428)	0.679
Nodal metastases (positive vs. negative)	1.778(1.227–2.578)	0.002	1.889(1.298–2.748)	0.001	1.44(0.902–2.299)	0.127
Primary tumor differentiation (poor vs. well to moderate)	1.158(0.779–1.721)	0.467			1.581(0.973–2.57)	0.064
Timing of metastasis (synchronous vs. metachronous)	0.74(0.49–1.118)	0.153			0.713(0.427–1.189)	0.195
RECIST response (SD/PD vs. PR)	1.097(0.76–1.583)	0.621			0.988(0.618–1.581)	0.960
Preoperative CEA (> 5 vs. ≤ 5 ng/mL)	1.333(0.922–1.927)	0.127			0.576(0.332–1.001)	0.051
Preoperative CA19-9 (> 35 vs. ≤ 35 U/mL)	1.083(0.714–1.643)	0.708			0.768(0.458–1.289)	0.318
Postoperative adjuvant therapy (yes vs. no)	1.539(1.034–2.291)	0.033	1.578(1.057–2.356)	0.026	1.557(0.997–2.431)	0.051
TBS (> 3 vs. ≤ 3)	1.18(0.8-1.741)	0.404			1.66(0.966–2.854)	0.067
Tumor distribution (bilobar vs. unilobar)	1.413(0.99–2.018)	0.057			1.652(1.048–2.603)	0.031
Postoperative chemotherapy (yes vs. no)	1.043(0.684-1,591)	0.845			1.233(0.716–2.123)	0.450

HR, hazard ratio; CI, confidence interval; RFS, recurrence-free survival;, OS, overall survival; RECIST, Response Evaluation Criteria in Solid Tumor; SD, stable disease; PD, progressive disease; PR, partial response; CEA, carcinoembryonic antigen; CA19-9, carbohydrate antigen 19 − 9; TBS, tumor burden score

For patients who did not receive NAC, the univariate and multivariate analyses of RFS and OS are summarized in Table 4. Univariate analysis revealed that positive nodal metastases, preoperative CEA > 5 ng/mL, TBS > 3, and bilobar disease were associated with unfavourable RFS. Multivariate analysis showed that nodal metastasis was positive (HR 2.083; 95% CI 1.24–3.50; *P* = 0.006), TBS > 3 (HR 1.175; 95% CI 1.049–1.317; *P* = 0.024) and bilobar disease (HR 1.786, 95% CI 1.037–3.078 *P* = 0.045) were also independent predictive factors for unfavourable RFS. Univariate analysis revealed that tumor site in the rectum, positive nodal metastases, preoperative CEA > 5 ng/mL and TBS > 3 were associated with unfavourable OS. Multivariate analysis showed that positive nodal metastases (HR 2.174; 95% CI 1.130–4.182; *P* = 0.020) and preoperative CEA > 5 ng/mL (HR 2.026; 95% CI 1-4.104; *P* = 0.050) were independent predictive factors for unfavourable OS.

**Table 4:** Univariate and multivariate analyses of prognostic factors for overall survival in patients without neoadjuvant chemotherapy

Characteristics	RFS				OS			
	Univariable		Multivariable		Univariable		Multivariable	
	HR(95% CI)	*P* value	HR(95% CI)	*P* value	HR(95% CI)	*P* value	HR(95% CI)	*P* value
Age (> 60 vs. ≤60 years)	0.967(0.631–1.483)	0.879			1.238(0.729–2.101)	0.430		
Sex (male vs. female)	1.081(0.695–1.682)	0.730			0.978(0.567–1.687)	0.937		
Primary tumor location (rectum vs. colon)	1.16(0.746–1.805)	0.510			1.754(1.030–2.987)	0.039	1.508(0.854–2.665)	0.157
Primary T stage (4 vs. 1–3)	0.985(0.627–1.549)	0.946			0.671(0.391–1.153)	0.149		
Nodal metastases (positive vs. negative)	2.243(1.356–3.708)	0.002	2.083(1.24–3.50)	0.006	2.066(1.108–3.853)	0.023	2.174(1.130–4.182)	0.020
Primary tumor differentiation (poor vs. well to moderate)	1.266(0.773–2.07)	0.349			1.104(0.582–2.096)	0.762		
Timing of metastasis(synchronous vs. metachronous)	0.88(0.57–1.359)	0.565			0.777(0.456–1.324)	0.343		
Preoperative CEA (> 5 vs. ≤ 5 ng/mL)	1.876(1.146–3.073)	0.012	1.553(0.938–2.572)	0.087	2.643(1.322–5.281)	0.006	2.026(1-4.104)	0.050
Preoperative CA19-9 (> 35 vs. ≤ 35 U/mL)	1.434(0.911–2.258)	0.119			0.576(0.332–1.001)	0.051		
Postoperative adjuvant therapy (yes vs. no)	1.337(0.934–1.916)	0.113			1.26(0.793-2.00)	0.328		
TBS (> 3 vs. ≤ 3)	1.893(1.236–2.899)	0.003	1.175(1.049–1.317)	0.024	1.736(1.022–2.949)	0.041	1.646(0.941–2.88)	0.081
Tumor distribution (bilobar vs. unilobar)	2.159(1.293–3.607)	0.003	1.786(1.037–3.078)	0.045	1.258(0.633–2.497)	0.512		
Postoperative chemotherapy (yes vs. no)	1.189(0.745–1.899)	0.467			1.185(0.672–2.090)	0.557		

HR, hazard ratio; CI, confidence interval; RFS, recurrence-free surviva;, OS, overall survival; CEA, carcinoembryonic antigen; CA19-9, carbohydrate antigen 19 − 9; TBS, tumor burden score

## Discussion

With the popularization of modern therapy technology and neoadjuvant therapy, the predictive value of traditional scoring systems has become limited[10]. The pathological TBS system has been proposed recently and has advantages over traditional CRS in predicting prognosis for patients with CRLM [11]. Preoperative TBS has been proven to have a prognostic ability similar to that of pathological TBS [17]. Although studies in Kazunari Sasaki et al. have shown that pathological TBS is suitable for predicting OS after NAC for CRLM, there is a lack of description of RFS [11]. In the current study, we evaluated the prognostic value of the pathological TBS system in patients who did or did not receive NAC. The data revealed that patients who received NAC presented higher TBS and lower median OS and RFS than patients who did not receive NAC. In addition, the survival curve showed that TBS only has discriminatory ability for OS and RFS in patients who did not receive NAC, and the survival curves of OS and RFS stratified by TBS for patients who received NAC cannot be distinguished. Moreover, in multivariate Cox analyses, pathological TBS was identified as an independent risk factor for RFS in patients who did not receive NAC.

Our data suggested that the TBS distribution of patients who received NAC was generally higher than that of patients who did not receive NAC (Fig. 1a and b), which can be explained by the following reasons. First, previous studies have indicated that patients with MSKCC-CRS 3–5 are recommended for NAC [18, 19] to improve their prognosis. Second, based on ESMO consensus guidelines and Chinese guidelines for the management of patients with colorectal liver metastatic cancer, patients with high CRS should receive NAC [13, 20]. Moreover, there were many patients whose initially unresectable CRLM tumor became resectable due to NAC. According to ESMO consensus guidelines [20], when complete macroscopic resection is feasible while maintaining at least a 30% future liver remnant (FLR) or a remnant liver to body weight ratio > 0.5 (e.g., > 350 g of liver per 70 kg patient), liver metastases should be considered technically resectable. Therefore, initially unresectable patients always have higher TBS. In addition, recent research suggested that NAC was more frequently administered in the TBS-high group [21]. As such, patients who received NAC generally have a heavier tumor burden, reflecting the right shift distribution of TBS.

A previous study indicated that TBS can predict the prognosis of CRLM [22]. However, our study found that TBS has prognostic value only in patients who did not receive NAC but not in patients who received NAC. Previous studies have also shown that MSKCC-CRS does not play a significant role in the prognosis of patients receiving NAC [10, 23]. It can be explained by two aspects. First, previous studies have shown that up to 6.07% of patients with liver metastases achieve a complete pathological response after NAC [24–26]. Therefore, because of the possible shrinkage and decreased number of tumors resulting from NAC, pathological TBS based on postoperative specimens may underestimate the initial tumor burden. Second, a recent study demonstrated that patients with a high TBS can gain a survival benefit from NAC [21]. Therefore, the prolonged survival of patients with high TBS may neutralize the adverse influence of high TBS, leading to the narrowed difference between the two groups. Interestingly, further subgroup analysis found that TBS did not predict the prognosis regardless of the chemotherapy response or the chemotherapy regimen. The results indicated that TBS is more likely to be an inherent indicator reflecting survival outcome to systemic treatment, regardless of how effective the chemotherapy and regimen are. Although Kazunari Sasaki et al. found a trend in predicting the prognosis of patients with progressive disease (PD)/ stable disease (SD) and partial response (PR), the study did not provide evidence of a significant difference [11]. Therefore, the prognostic role of TBS in different chemotherapy responses remains to be further studied.

Based on the above results, TBS could be applied as an important parameter to predict the RFS and OS of patients who did not receive NAC but not in patients who received NAC. Previous studies have found that the TBS obtained by preoperative imaging is similar to that obtained by pathology in terms of predictive ability in patients who received NAC, and other studies have shown that the change in tumor burden due to chemotherapy is also a good predictor [27]. Based on this, we speculate that the prognostic ability of pre-NAC TBS may be less susceptible to NAC. Therefore, further exploration of the predictive ability of pre-NAC for the prognosis of patients or study of the change rate of TBS before and after NAC may help to improve the predictive ability of TBS in patients receiving NAC.

We acknowledge that limitations exist in regard to this study. First, the limitations of retrospective analyses and a single institution apply. Therefore, data from other institutions would be beneficial to further validate our hypothesis and the external validity of our predictive models. Second, RAS and BRAF are considered significant prognostic factors and thus have been widely applied in clinical practice [28, 29]. However, we did not include these gene statuses in the analysis because of unavailability. Moreover, perioperative chemotherapy may be relevant for early recurrence and prognosis, but this was not analysed in the current study. In addition, another study showed that the ability of imaging-based TBS to preoperatively predict prognosis was comparable to that of pathology-based TBS [17]. Therefore, future studies are required to investigate whether imaging-based TBS before chemotherapy is superior to pathological TBS in terms of predicting the prognosis of patients who receive NAC so that individualized treatment regimens can be determined before surgery.

## Conclusion

Pathological TBS can be applied to predict the RFS and OS of patients suffering from CRLM who did not receive NAC. However, pathological TBS might not be powerful enough to predict the prognosis of patients who did receive NAC.

## Data Availability

The datasets used and/or analyzed during the current study available from the corresponding author on reasonable request.
